# Ein innovativer Regulierungsansatz zur Belebung des Wettbewerbs im Schienenpersonenfernverkehr

**DOI:** 10.1007/s10273-021-2939-9

**Published:** 2021-06-22

**Authors:** Alexander Eisenkopf, Andreas Knorr

**Affiliations:** 1 Zeppelin-LS f. Wirtschafts- u. Verkehrspolitik, Zeppelin Universität Friedrichshafen, Am Seemooser Horn 20 D, 88045 Friedrichshafen, Deutschland; 2 Verwaltungswissenschaften Speyer, Deutsche Universität für, Freiherr-vom-Stein-Straße 2, 67346 Speyer, Deutschland

**Keywords:** L41, L51, L92

## Abstract

Im Schienenpersonenfernverkehr bestehen trotz Bahnstrukturreform weiterhin hohe Markteintrittsbarrieren. Der Wettbewerb im Nah- und Schienengüterverkehr ist im Vergleich dazu bereits besser in Gang gekommen. Die Autoren schlagen einen innovativen Regulierungsansatz vor, um den intramodalen Wettbewerb im Schienenpersonenfernverkehr zu beleben. Mitbewerbern der Deutschen Bahn soll nicht nur der offene Zugang zu Gleisen, sondern auch zu Zügen und Wagen — also Sitzplatzkontingenten — ermöglicht werden.

## Abstract (en)

Regulatory efforts to introduce effective intramodal competition to the rail sector, in particular to the long-distance intercity market segment, have so far achieved only very limited success in most countries. Germany is a case in point. Even 25 years after the fundamental 1994 ‘rail structure reform’, which eliminated the legal network monopoly of the former Deutsche Bundesbahn (DB), the combined market share of all competitors for long-distance passenger services remains stuck at “significantly below one per cent” (Bundesnetzagentur 2018, 22). This figure stands in stark contrast to the competitors’ market shares of some 26% for short-distance (regional) services — where a competition for the market regime has been established — and around 50% of the rail cargo segment — which is governed by an open access regime. In this paper, we make the case for an alternative regulatory regime to substantially increase effective intramodal competition in the intercity rail passenger market. Our proposal addresses, and helps to overcome, the two major entry barriers faced by competitors: the massive investment needs in rolling stock on the one hand and reliable infrastructure access on the other.

Historisch gesehen gehört der Schienenverkehr zu den am stärksten regulierten Wirtschaftssektoren in Europa. Dies liegt zum einen an den strukturellen Voraussetzungen dieses Sektors mit der Schieneninfrastruktur als natürlichem Monopol, zum anderen aber auch an den politisch relevanten gemeinwirtschaftlichen Verpflichtungen in diesem Feld. Hinzu kommt das Potenzial für Marktmachtmissbrauch aufgrund von Infrastrukturengpässen und des Fehlens eines effektiven intermodalen Wettbewerbs (Nash, 2013). Die traditionelle Regulierung des Bahnsektors deckt – unabhängig von den spezifischen Eigentumsverhältnissen – verschiedene Bereiche ab: Markteintritt und -austritt, Regeln für den Infrastrukturzugang, Preisgestaltung, Technologie (insbesondere zur Gewährleistung der Interoperabilität) und in jüngerer Zeit auch Fragen der Dienstleistungsqualität und des Verbraucherschutzes (Finger und Messulam, 2015).

Trotz umfassender Bemühungen zur Einführung und Belebung des intramodalen Wettbewerbs im Schienenverkehr in den meisten europäischen Ländern war die Politik der Europäischen Union nur bedingt erfolgreich. Dies gilt insbesondere für den Schienenpersonenfernverkehr (SPFV), wofür Deutschland ein Paradebeispiel ist. Auch ein Vierteljahrhundert nach der Bahnstrukturreform von 1994 ist die Deutsche Bahn AG trotz eines offenen Zugangsregimes nach wie vor de facto Monopolist im SPFV. Im Nahverkehr hatte sie im letzten vollen Geschäftsjahr vor Ausbruch der COVID-19-Pandemie gemessen an den Zugkilometern immer noch einen Marktanteil von 74 %, während beim Schienengüterverkehr die Wettbewerber mittlerweile mehr als die Hälfte der Kunden bedienen (gemessen in Tonnenkilometern) (Monopolkommission, 2020).

**Prof. Dr. Alexander Eisenkopf** hat den Zeppelin-Lehrstuhl für Wirtschafts- und Verkehrspolitik an der Zeppelin Universität Friedrichshafen inne.

**Prof. Dr. Andreas Knorr** ist Professor für Volkswirtschaftslehre, insbesondere für Wirtschafts- und Verkehrspolitik, an der Deutschen Universität für Verwaltungswissenschaften Speyer.

Während im Nah- und im Güterverkehr Wettbewerb durchaus in Gang gekommen ist, bleibt die Entwicklung im SPFV absolut unbefriedigend. Daher wird hier ein alternativer Regulierungsansatz skizziert, der den intramodalen Wettbewerb im SPFV in Deutschland beleben soll. Der Vorschlag stellt eine radikale Abkehr von der traditionellen Doktrin dar, den konkurrierenden Bahnbetreibern diskriminierungsfreien Zugang zur Infrastruktur zu gewährleisten. Der Ansatz basiert vielmehr auf einer Neudefinition dessen, was als monopolistischer Engpass zu betrachten ist: Als kritische Infrastrukturkomponente auf stark ausgelasteten, aber monopolistisch bedienten Fernstrecken ist dies nicht mehr der offene Zugang zu den Gleisen, sondern der Zugang zu den Zügen und Wagen, die vom etablierten Betreiber auf diesen Strecken betrieben werden. Es geht also um den nichtdiskriminierenden Zugang von Konkurrenten zum Sitzplatzangebot des Monopolisten.

## Theoretische Grundlagen

### Wertschöpfungskette im Schienenverkehr

Ausgangspunkt für ein angemessenes regulatorisches Verständnis des Schienenverkehrs ist die Unterscheidung zwischen Infrastruktur- (bzw. Netz-)Leistungen (d.h. Bau, Betrieb und Instandhaltung der Schieneninfrastruktur) einerseits und Transportleistungen (d.h. Personen- und Gütertransport) andererseits. Dieser Ansatz entspricht der langjährigen Arbeitsteilung zwischen spezialisierten Infrastrukturbetreibern und Dienstleistungsanbietern für alle anderen Verkehrsträger.

Bei der Eisenbahn wird häufig noch die Koordination des Netzzugangs und der Netzauslastung (d.h. die Zuweisung von Gleisen und Bahnhofsinfrastrukturen im Sinne von zeitdefinierten Nutzungsrechten – „Slots" – für Zugfahrten) und damit implizit auch die Fahrplan- und Terminkoordination als dritte Ebene angesprochen. In einem solchen dreistufigen Modell ist die Steuerungsebene vom originären Aufgabenbereich des Netzbetreibers zu trennen, um diskriminierungsfreien Netzzugang zu gewährleisten (grundlegend Knieps, 1996).

## Vertikale Trennung vs. diskriminierungsfreier Zugang

Theoretisch gibt es verschiedene Möglichkeiten, intramodalen Wettbewerb im Eisenbahnsektor anzureizen. Neben der strikten Trennung der Wertschöpfungsebenen Netz und Transport ist es grundsätzlich auch möglich, intramodalen Wettbewerb bei gleichzeitiger vertikaler Integration der beiden Wertschöpfungsebenen über diskriminierungsfreien Netzzugang (Open Access) zu gewährleisten. Dazu bedarf es im zweiten Fall jedoch einer umfassenden und intensiven Regulierung (Seabright, 2003). Im Falle einer vertikalen Desintegration von Netz- und Zugbetrieb erfolgt dagegen eine vollständige eigentumsrechtliche Entflechtung, oder zumindest die institutionelle Trennung der entsprechenden Stufen der Wertschöpfungskette. Diese Lösung des Netzzugangsproblems ist aus regulatorischer Sicht besonders interessant, denn sie beseitigt jeglichen Anreiz für den Infrastrukturbetreiber, bei der Zuteilung von Infrastrukturnutzungsrechten seine eigenen Transportabteilungen zu begünstigen (Nash, 2013). Umgekehrt haben vertikal integrierte Eisenbahnverkehrsunternehmen starke Anreize, aktuelle sowie potenzielle Wettbewerber im Transportgeschäft zu diskriminieren (Heddrich, 1996).

Für eine ausgewogene Bewertung ist es aber auch notwendig, die möglichen wirtschaftlichen Nachteile einer vertikalen Trennung zu diskutieren. Sie resultieren vor allem aus dem möglichen Verlust von Verbundvorteilen und aus höheren Transaktionskosten. Auch innerhalb eines vertikal integrierten Eisenbahnunternehmens existieren relevante Koordinationskosten, die typischerweise erheblich unterschätzt werden (Laaser, 1994). Ökonomische Verbundvorteile können durchaus auch durch symbiotische organisatorische Arrangements und hybride Koordinationsschemata nutzbar gemacht werden. Darüber hinaus sind einige der wirtschaftlichen Vorteile der vertikalen Integration gleichbedeutend mit wettbewerblichen Nachteilen für Eisenbahnbetriebsunternehmen ohne Netzeigentum.

Was Deutschland betrifft, ist die vollständige vertikale Trennung von Netz und Diensten bisher weder im Eisenbahnsektor noch in den meisten anderen Netzsektoren (mit Ausnahme der Elektrizitätswirtschaft und teilweise der Gaswirtschaft) erreicht worden. Dies mag jedoch nicht auf wirtschaftliche Nachteile oder technische Undurchführbarkeit zurückzuführen sein, sondern ist in erster Linie das Ergebnis der Dominanz des Status quo und des Einflusses der Netzeigentümer auf den politischen Entscheidungsprozess.

Die ordnungspolitische Alternative zur vertikalen Trennung von Schienennetz und Dienstleistungen stellt der diskriminierungsfreie Netzzugang Dritter dar. Bei diesem Ansatz wird die traditionelle institutionelle Integration von Infrastruktur und Transportdienstleistungen beibehalten. Der Infrastruktursektor ist jedoch verpflichtet, Eisenbahnunternehmen ohne eigene Netzinfrastruktur einen diskriminierungsfreien Netzzugang zu gewähren. Um dies umzusetzen, sind zumindest eine buchhalterische Trennung zwischen Infrastruktur- und Verkehrsleistungen sowie Neutralität bei der Trassenvergabe unabdingbar (Knieps, 2015). Weiterhin müssen Gesetzgeber und zuständige Regulierungsbehörde detaillierte Vorgaben für den Zugang zur Infrastruktur machen und den Netzzugang überwachen. Im Kartellrecht wird das Problem des diskriminierungsfreien Netzzugangs mit Hilfe der Essential-Facilities-Doktrin adressiert. Dieses Rechtskonzept kommt zur Anwendung, wenn sich Netze oder andere wesentliche Einrichtungen in den Händen vertikal integrierter Unternehmen befinden (Knieps, 1996).

## Wettbewerb auf dem Markt vs. Wettbewerb um den Markt

Diskriminierungsfreier Marktzugang oder Open Access basiert immer auf der Annahme, dass der relevante Markt für Transportdienstleistungen profitable Geschäftsmodelle ermöglicht. Die Idee ist, dass diese Verkehrsdienste ohne Subventionen betrieben werden können. Daher muss ein Interesse der Eisenbahnverkehrsunternehmen am Markteintritt bestehen, damit intramodaler Wettbewerb überhaupt wirksam werden kann. So hat die Öffnung des Schienengüterverkehrsmarkts der EU zwar zu zahlreichen Markteintritten geführt, jedoch nicht zu einer Veränderung der Wettbewerbssituation im Einzelwagenverkehr. Dieses Geschäftsmodell ist für die Konkurrenten der etablierten Bahnbetreiber offensichtlich zu unattraktiv. Hier ist – im Gegensatz zum Markt für Ganzzugverkehre – ohne öffentliche Subventionen anscheinend nicht mit einem Markteintritt privater Anbieter zu rechnen.

Ein Grund für den stark unterentwickelten Wettbewerb im SPFV mag sein, dass die Öffnung in diesem Marktsegment im Vergleich zum Schienengüterverkehr immer wieder verschoben und damit erheblich verzögert wurde. Für den deutschen Markt trifft dies jedoch nicht zu, da der Zugang im SPFV bereits 1994 und damit lange vor der allgemeinen Marktöffnung auf EU-Ebene gesetzlich garantiert wurde (van de Velde, 2015). Vielmehr dürfte die Marktstruktur auch auf die relativ geringe Rentabilität dieses Markts und die schwierigen Wettbewerbsbedingungen zurückzuführen sein (Feuerstein, Busacker und Xu, 2018). Ein weiterer Grund für mangelnden Wettbewerb in Europa insgesamt dürfte darin liegen, dass ausländische staatliche Eisenbahngesellschaften keine potenziellen Konkurrenten der nationalen etablierten Bahnen sein wollen und können, da sie im grenzüberschreitenden Verkehrssegment weiterhin eng zusammenarbeiten und daher auf ihrem jeweiligen „Heimatgebiet" in den nationalen Eisenbahnsystemen nicht in einen intensiven Wettbewerb gegeneinander treten.

Wenn ein Markt für neue Wettbewerber nicht attraktiv ist oder gar nicht bedient wird, weil dauerhafte Verluste nicht vermieden werden können, scheint es eine gute Alternative zu sein, Wettbewerb um den Markt zu etablieren, um die Erbringung der im Sinne der Daseinsvorsorge gewünschten Dienstleistungen sicherzustellen. Diese Idee geht auf Demsetz (1968) zurück, der bereits in den 1960er Jahren zeigte, wie die Marktkräfte genutzt werden können, um den Anbieter mit dem geringsten Bedarf an öffentlichen Subventionen für die Leistungserbringung auszuwählen. Kommt Wettbewerb auf einem Verkehrsmarkt nicht zustande, gibt es somit starke Argumente für eine wettbewerbsorientierte Ausschreibung von Transportdienstleistungen, vor allem aufgrund der damit verbundenen Anreize zur Steigerung von Effizienz und Qualität, wie z. B. im deutschen Schienenpersonennahverkehr zu beobachten ist.

## Intramodaler Wettbewerb und produktive Effizienz

Vor der Bahnreform 1994 wurden die Eisenbahnen in West- und Ostdeutschland als vertikal integrierte, staatliche Unternehmen organisiert und geführt, die keinem intramodalen Wettbewerb ausgesetzt waren. Darüber hinaus wurden und werden Eisenbahninfrastrukturen und Schienenpersonenverkehr wie in praktisch allen anderen Industrieländern jahrzehntelang stark subventioniert (European Environmental Agency, 2007). Es ist daher höchst unwahrscheinlich, dass integrierte Eisenbahnunternehmen wie die Deutsche Bahn mit höchstmöglicher Effizienz arbeiten. Offensichtlich ist, dass es im Vergleich zu intermodalen Wettbewerbern wie dem Lkw- oder dem Luftverkehr seit Jahrzehnten eine Innovationsschwäche gibt; Marktschwächen und die daraus resultierenden schlechten wirtschaftlichen Ergebnisse der Bahnen werden jedoch in der (deutschen) verkehrspolitischen Diskussion häufig als Folge von intermodalen Wettbewerbsverzerrungen und mangelnden Investitionen des staatlichen Eigentümers insbesondere in die Infrastruktur angesehen (Eisenkopf, 2004).

Auf Basis des Modells der drei Wertschöpfungsebenen ist es möglich, Wettbewerb auf dem Markt für Eisenbahnverkehrsleistungen zu organisieren. Aus dem Markteintritt neuer Wettbewerber sollten sich Effizienzsteigerungen und eine nachhaltige Verbesserung der Marktposition der Eisenbahnen gegenüber ihren intermodalen Wettbewerbern ergeben (Nash, 2013). Dahinter steht die Überlegung, dass der potenzielle Marktzutritt den Druck auf etablierte Betreiber erhöht, die Qualität zu verbessern und Kosten zu senken. Intramodaler Wettbewerb wird dabei als wesentlich wirksamer hinsichtlich Kosteneffizienz und Marktorientierung als die vorhandene intermodale Konkurrenz angesehen.

Allerdings hat sich die Öffnung der Schienenverkehrsmärkte für neue Transportdienstleister in der europäischen Praxis als relativ schwierig erwiesen, nicht nur wegen des (verständlichen) politischen Widerstands der etablierten staatseigenen Bahnen (Incumbents), sondern auch wegen des politischen Interesses ihrer staatlichen Eigentümer an der Aufrechterhaltung des Status quo. Eine rationale Schienenverkehrspolitik sollte sich jedoch nicht auf den Schutz eines dominanten Incumbent konzentrieren, sondern auf die Förderung des Schienenverkehrssystems als Ganzes. Vor allem dank der Beharrlichkeit der EU sind heute zumindest rechtlich die Voraussetzungen für einen nachhaltigen intramodalen Wettbewerb auf den Schienenverkehrsmärkten in allen EU-Mitgliedstaaten geschaffen worden (UNECE, 2017). Trotzdem hat sich der intramodale Wettbewerb im Schienenpersonenfernverkehr nicht wie erwartet entfaltet. Dies gilt insbesondere auch für Deutschland (Cherbonnier et al., 2018; van de Velde, 2015).

## Open-Access-Regime in der Praxis wenig erfolgreich

Die Steigerung der Effizienz und Wettbewerbsfähigkeit der Eisenbahn zur Förderung des Marktanteils der umweltfreundlichen Schiene ist eines der wichtigsten verkehrspolitischen Ziele der EU und der nationalen Verkehrspolitiken, um die aktuellen und zukünftigen Herausforderungen der Verkehrsmärkte, insbesondere die steigende Nachfrage im Personenfern- und Güterverkehr, zu bewältigen (Nash, 2006). In dicht besiedelten Ländern mit einer diversifizierten industriellen Basis wie Deutschland sind Bahnbetreiber in bestimmten Marktsegmenten intermodalem Wettbewerb ausgesetzt, wenn auch in unterschiedlichem Ausmaß. Dies gilt z.B. für den Nahverkehr in großen Ballungsräumen – im Wettbewerb mit dem motorisierten Individualverkehr – und zunehmend auch im SPFV durch das Aufkommen von Billigfluglinien. Ab 2013 traten in Deutschland Fernbusverkehre als neue Wettbewerber auf. Der intermodale Wettbewerb durch diese neuen Player hat die Deutsche Bahn zwar gezwungen, die Preise zu senken und den Fahrgastkomfort auf einigen Fernverkehrsstrecken zu erhöhen, der Gesamteffekt ist aber insgesamt noch bescheiden (detailliert: Knorr und Lueg-Arndt, 2016).

Vor diesem Hintergrund sollte die Intensivierung des intramodalen Wettbewerbs der Schlüssel zu signifikanten Fortschritten sein, um die intermodale Wettbewerbsfähigkeit der Eisenbahnen zu verbessern. Dementsprechend und in Übereinstimmung mit den theoretischen Überlegungen war die Neugestaltung des bestehenden Regulierungsrahmens auf EU-Ebene der Ausgangspunkt zur Belebung des intramodalen Wettbewerbs im SPFV. Ausgehend von der Richtlinie EWG 91/440 der Europäischen Kommission wurde die Marktöffnung durch vier Eisenbahnliberalisierungspakete vorangetrieben. In der EU wird der Markt für inländische Schienenpersonenfernverkehrsdienste ab 2022 vollständig liberalisiert sein, wobei unbegrenzte Kabotage erlaubt sein wird. Damit hat die EU eine klare Regelung für den offenen Zugang zu diesem Marktsegment verabschiedet (Perennes, 2016). Marktzugangsregeln für den Schienenpersonenfernverkehr im Einklang mit dem EU-Rahmen sind bereits in mehreren derzeitigen EU-Mitgliedstaaten (plus Großbritannien als vorheriges Mitglied) gängige Praxis (Cherbonnier et al., 2018). Tatsächlicher Wettbewerb auf der Basis von Open-Access-Modellen bleibt in diesem Marktsegment in Europa aber ein seltenes Phänomen (Bergantino, Capozza und Capurso, 2015), wobei der italienische Hochgeschwindigkeitsbahnmarkt (wo der Newcomer Italo – Nuovo Trasporto Viaggiatori (NTV) ein Drittel des Markts erobert hat) die einzige relevante Ausnahme darstellt (Desmaris, 2016). In allen anderen Ländern, die sich für ein offenes Zugangsregime entschieden haben (d.h. in der Tschechischen Republik, Österreich, Schweden, Großbritannien und Deutschland), kam es nur auf wenigen Strecken zu Markteintritten von Neueinsteigern, die nur einen begrenzten kommerziellen Erfolg hatten.

In Deutschland wurden zwischen 2000 und 2010 insgesamt zehn Markteintrittsversuche unternommen, allerdings auf sehr bescheidenem Niveau hinsichtlich der Frequenzen und der Sitzplatzkapazität, von denen sechs nach relativ kurzer Zeit wieder eingestellt wurden (Séguret, 2009). Abgesehen von einigen wenigen marginalen Anbietern war vor dem Einbruch infolge der Corona-Krise nur Flixtrain auf dem inländischen Fernverkehrsmarkt aktiv, allerdings auch nur auf wenigen Strecken und mit geringer Frequenz (Flixtrain, 2019). Angesichts dieser Befunde kann man nicht wirklich von einem wirksamen Wettbewerb im Schienenpersonenfernverkehr sprechen, wie die Monopolkommission in ihrem letzten Sondergutachten zum Schienenverkehrsmarkt noch einmal ausdrücklich festgestellt hat (Monopolkommission, 2019).

Blickt man ins Ausland, zeigt die Etablierung eines Wettbewerbs im SPFV über einen Markt für Ausschreibungen vor allem in Großbritannien, dass auch diese alternative Marktordnung nicht reibungslos funktioniert. Zwar wurde auch dort gelegentlich ein offener Zugang für ausgewählte SPFV-Dienste gewährt, doch werden diese in ihrer überwiegenden Mehrzahl derzeit von den 16 Franchiseunternehmen erbracht. Festzuhalten ist somit, dass das Franchising-System in Großbritannien zwar mit starkem Verkehrswachstum verbunden war, die erzielten Kostensenkungen aber dennoch unbefriedigend geblieben sind. Darüber hinaus ist immer wieder das Problem des Winner‘s Curse aufgetreten, wenn Bietende aufgrund zu optimistischer Prognosen kommerziell nicht erfolgreich waren. Ein Vergleich der jeweiligen Wettbewerbs- und Regulierungsregime in Großbritannien, Schweden und Deutschland zeigt, dass nicht abschließend geklärt ist, welches das bessere Modell der Marktorganisation ist. Das stärkere Wachstum der Transportdienstleistungen sowohl im Personen- als auch im Güterverkehrsmarkt scheint jedenfalls mit einem höheren Bedarf an staatlicher Subventionierung verknüpft zu sein (Nash, Nilsson und Link, 2013).

Zusammenfassend lässt sich festhalten, dass das Gesamtbild gemischt ist: Weder ein offener Zugang noch der Wettbewerb um den Markt bilden eine „Wunderwaffe“ für die Etablierung eines effektiven intramodalen Wettbewerbs im SPFV. Insbesondere fällt auf, dass – anders als in der Luftfahrt – im SPFV bisher kein nennenswerter Markteintritt von Low-Cost-Eisenbahnunternehmen stattgefunden hat. Tatsächlich wurde dieses Geschäftsmodell – mit Ausnahme des deutschen Flixtrain sowie der tschechischen Anbieter RegioJet und LEO Express – im SPFV bislang kaum erprobt.

## Zugang zum Sitzplatzinventar

### Konzeptionelle Überlegungen

Die traditionellen Ansätze für eine Marktöffnung haben nicht zu relevantem intramodalen Wettbewerb im

Schienenpersonenfernverkehr geführt, weil die potenziellen Wettbewerber trotz der rechtlichen Marktöffnung aufgrund der institutionellen Rahmenbedingungen des Schienenverkehrsmarkts bisher nicht in der Lage sind, eine kritische Masse an Sitzplätzen auf den betreffenden Städtepaaren darzustellen. Ein konkurrierender Punkt-zu-Punkt-Verkehr mit zwei oder drei Zugpaaren pro Tag führt noch nicht zu wirksamem Wettbewerb auf der betroffenen Strecke, außer im ertragsschwachen Segment der preisbewussten Freizeitreisenden. Zudem muss sichergestellt werden, dass Wettbewerber nicht nur auf einzelnen Strecken konkurrieren, sondern in der Lage sind, ein möglichst breites Netz zu bedienen, um eine größere Zahl von Städtepaaren verbinden zu können ähnlich dem Angebot von Low-Cost-Airlines und dem Intercity-Busverkehr von Flixbus.

Zu den institutionellen und ökonomischen Rahmenbedingungen, die als Markteintrittsbarrieren für Newcomer im SPFV wirken, gehören:

- Hoher Kapitalbedarf für spezifische Investitionen in rollendes Material;
- begrenztes Angebot an Schienenfahrzeugen aufgrund hoher Auslastung der europäischen Hersteller und erheblicher Marktzutrittsschranken gegenüber Nicht-EU-Schienenfahrzeugherstellern aus Korea, Japan und China und eines rudimentären Leasing- und Gebrauchtwagenmarkts;
- enge Beziehungen zwischen den Rollmaterialherstellern und den etablierten Bahnbetreibern, die den Anreiz für die Hersteller verringern können, Verträge mit Neueinsteigern abzuschließen;
- zunehmender Mangel an Triebfahrzeugführer:innen, Rangierer:innen und Personal für Stellwerke;
- anhaltende Schwierigkeiten bei der Erlangung eines diskriminierungsfreien Zugangs zu den Auskunftsdiensten der Deutschen Bahn (um Fahrplaninformationen der breiten Öffentlichkeit zugänglich zu machen).

Da diese Probleme kurzfristig kaum überwindbar erscheinen, schlagen wir als Alternative ein ergänzendes einfaches, aber potenziell sehr wirksames neues Regulierungssystem vor, den regulierten Zugang zum Sitzplatzangebot des marktbeherrschenden Anbieters. Wir sind der Überzeugung, dass die verfügbare Sitzplatzkapazität aus Sicht von Newcomern, die Langstreckendienste anbieten wollen, derzeit faktisch der wichtigste monopolistische Engpass und somit die relevante wesentliche Einrichtung ist.

Dabei ist zunächst einmal von großer Bedeutung, dass die durchschnittliche Zugauslastung im Personenfernverkehr in Deutschland 2019 (vor Corona) bei nur 56,1 % lag. Zwar ist die Auslastungsquote gegenüber 2010 (48 %) vor allem aufgrund des verstärkten intermodalen Wettbewerbs durch den Busfernverkehr und des daraus resultierenden aggressiven Preiskampfes etwas gestiegen, doch stagnierte sie in den vergangenen beiden Jahren (bereits vor dem Absturz im Zuge der Corona-Krise) (Deutsche Bahn AG, 2020). Zwar ist die Auslastung in den Hauptreisezeiten und zu Spitzenzeiten am Tage deutlich höher, aber am Ende bleibt im Durchschnitt immer noch fast die Hälfte der Sitze unverkauft. Außerdem sollte auch nicht übersehen werden, dass für den Fernverkehr in den Zügen der Deutschen Bahn keine obligatorische Sitzplatzreservierung im Voraus erforderlich ist; d. h. selbst wenn alle Plätze in einem Zug besetzt sind, können zusätzliche Fahrgäste einsteigen, solange genügend Stehplätze in einem Wagen vorhanden sind, um eine sichere Fahrt zu gewährleisten. Dementsprechend kann die effektiv nutzbare Kapazität eines Zuges während der Hauptreisezeiten 100 % der Sitzplatzkapazität bei weitem übersteigen.

Der vorgeschlagene regulierte Zugang zum Sitzplatzangebot des marktbeherrschenden Incumbent für Wettbewerber könnte in zwei Varianten umgesetzt werden:

- Entweder eröffnet die Regulierungsbehörde den Wettbewerbern den vollen Zugang zu einem bestimmten Prozentsatz aller Sitze in einer einzelnen Zugeinheit (z. B. 10 % oder 20 % aller in einem bestimmten Zug angebotenen Sitze) unter nicht diskriminierenden Bedingungen, d. h. auf der Grundlage der Grenzkosten pro Sitzplatz des etablierten Betreibers. Die Konkurrenten würden dann versuchen, diese Sitze im Wettbewerb zu den Fahrkartenangeboten des etablierten Betreibers direkt oder über Vermittler an potenzielle Reisende zu verkaufen.
- Alternativ könnte das Regulierungssystem so gestaltet werden, dass die Wettbewerber exklusiven Zugang zu einer bestimmten Zahl von Wagen in einem bestimmten Zug (mindestens einer) sowie das Recht erhalten, diese Kapazität unabhängig zu vermarkten.

### Codeshare-Vereinbarungen in der Luft und auf der Schiene

Der Vorschlag eines regulierten Netzzugangs zu Sitzplatzkapazitäten des monopolistischen Anbieters nimmt Anleihen bei dem in der Luftfahrtindustrie populären Modell des Code-Sharing. Hierbei planen zwei oder mehr Fluggesellschaften gemeinsam einen Flug und vermarkten diesen unter ihren jeweiligen Airline-

Kennungen und Flugnummern; der Flug wird allerdings nur von einer der Gesellschaften durchgeführt. Zur Umsetzung haben sich verschiedene Modelle wie die Blockplatz- oder die Free-Flow-Regelung etabliert. Es kommen auch Vereinbarungen zur Einnahmen- bzw. Gewinnaufteilung vor.

Codeshare-Agreements können hinsichtlich paralleler Flüge oder auch für Anschluss- und Zubringerdienste getroffen werden. Im zweiten Fall verbinden beide Fluggesellschaften ihre Netze miteinander, um Reiserouten zwischen Zielen anzubieten, die sonst nur von einem der Codeshare-Partner bedient würden. Codeshare-Vereinbarungen in der Luftverkehrsbranche sind von Ökonom:innen im Hinblick auf ihre Auswirkungen auf Flugkosten, Flugpreise, Vorteile für die Verbraucher und ihre möglichen kartellrechtlichen Auswirkungen sehr genau analysiert worden (stellvertretend: Yimga und Gorjidooz, 2019). In der Regel werden erhebliche Vorteile von Codeshare-Vereinbarungen für die beteiligten Fluggesellschaften aufzeigt. Auf der Kostenseite ergeben sich niedrigere Produktionskosten aufgrund einer besseren Kapazitätsauslastung; auf der Einnahmenseite dürfte ein weniger intensiver Preiswettbewerb ceteris paribus die Erlöse und letztendlich die Gewinne steigern.

Aus Sicht der Passagiere und des Kartellrechts sind die Vorteile von Codeshare-Vereinbarungen jedoch weniger klar. Tatsächlich bieten Codeshares auf Umsteigeverbindungen den Kund:innen Vorteile in Form von nahtlosen Reisemöglichkeiten für eine größere Zahl verfügbarer Städtepaare. Im Gegensatz dazu verringern Codeshare-Vereinbarungen bei parallelen Diensten häufig den Wettbewerb zwischen den Codeshare-Partnern, was tendenziell zu höheren Flugpreisen führt, wenn nicht ein gewisses Maß an intermodalem und vor allem intramodalem Wettbewerb (durch andere Fluggesellschaften, insbesondere Billigfluggesellschaften) aufrechterhalten werden kann. Dieses wettbewerbspolitische Problem tritt besonders dann zutage, wenn Codeshare-Vereinbarungen zur Aufteilung von Einnahmen und Gewinnen verwendet werden. Daher müssen die Kartellbehörden bei der Beurteilung dieser Vereinbarungen die potenziellen Vorteile des Codeshare für die Verbraucher:innen gegen das erhöhte Risiko von Absprachen zwischen den mittels Codeshare kooperierenden Fluggesellschaften abwägen.

Freiwillige Codeshare-Vereinbarungen gibt es in unterschiedlicher Form und seit langem auch im Eisenbahnsektor. Im Gegensatz zu den Codeshares der Fluggesellschaften werden sie jedoch ausschließlich für Anschlussleistungen genutzt und existieren in zwei grundlegenden Varianten: zwischen Bahnbetreibern aus verschiedenen Ländern und zwischen Bahnbetreibern und Fluggesellschaften im Zu- und Abbringerverkehr nach/von den Verkehrsflughäfen. So arbeiten die Eisenbahnverkehrsunternehmen bei der Erbringung grenzüberschreitender Eisenbahndienstleistungen seit langem eng zusammen. Diese Zusammenarbeit besteht nicht nur darin, dass nationale Züge der beteiligten Unternehmen auf internationalen Strecken eingesetzt werden, d. h. durch die gemeinsame Nutzung von Personal und Rollmaterial und die Gewährleistung des gegenseitigen Zugangs zur Infrastruktur in allen Ländern, die bedient oder durchfahren werden. Insbesondere verkaufen beide Unternehmen Fahrkarten für die jeweilige Verbindung, häufig zu differenzierten Tarifen für identische Streckenführungen, was Arbitrage-Möglichkeiten für Intermediäre wie Trainline eröffnet.

Ein weiteres praktisches Beispiel aus Deutschland ist die Vereinbarung zwischen der Lufthansa und der Deutschen Bahn über Lufthansa Express Rail. Dieses intermodale Angebot kombiniert ein Zugticket und ein Flugticket und gilt für ICE-Hochgeschwindigkeitszüge vom und zum Flughafen Frankfurt – dem Hauptdrehkreuz der Lufthansa –, um unwirtschaftliche kurze Zubringerflüge durch effizientere Zugverbindungen zu ersetzen. Das Angebot besteht derzeit zwischen 17 deutschen Großstädten, und es wird kontinuierlich auf mehr Züge ausgeweitet. Die Tickets werden direkt von Lufthansa verkauft, auch die jeweiligen Züge erhalten eine Lufthansa-Flugnummer. In jedem infrage kommenden Zug sind zwei Wagen – je einer in der ersten Klasse und zweiten Klasse – für Lufthansa Express Rail Passagiere reserviert. Deren Tickets enthalten zwar eine Sitzplatzzuweisung, aber alle nicht genutzten Sitze in diesen speziellen Wagen können auch von den eigenen Kunden der Deutschen Bahn in Anspruch genommen werden.

Ein weiteres verwandtes Beispiel ist das seit langem bestehende Rail&Fly-Tarifsystem der Deutschen Bahn. Es erlaubt (einigen) Fluggesellschaften den Verkauf von Bahntickets von jedem deutschen Bahnhof zu jedem deutschen Flughafen als zusätzliche Segmente der ausgestellten Flugtickets. Diese Regelung ist besonders attraktiv für ausländische Fluggesellschaften, die keinen eigenen inländischen Zubringerflug von und zu ihrem Gateway-Flughafen in Deutschland durchführen bzw. in Ermangelung von Verkehrsrechten nicht dazu berechtigt sind. Die Preise der jeweiligen Rail&Fly-Tarife unterscheiden sich je nach Fluggesellschaft erheblich, in Abhängigkeit vom Ergebnis ihrer Preisverhandlungen mit der Deutschen Bahn und der Preisstrategie der Fluggesellschaften. Rail&Fly-Tickets werden nicht von der Deutschen Bahn verkauft, sondern müssen während des Buchungsprozesses direkt bei der Fluggesellschaft hinzugebucht werden.

### Vor- und Nachteile eines regulierten Zugangs zum Sitzplatzangebot

Die innovative Open-Access-Regulierung, die einen marktbeherrschenden Incumbent verpflichten würde, seine unverkauften Sitzplatzkapazitäten für Wettbewerber zu öffnen, beseitigt grundlegende Probleme der bisher praktizierten Marktzugangsregulierung. Unter der traditionellen Regulierung blieben trotz der Absenkung bzw. Aufhebung rechtlicher Eintrittsbarrieren die wirtschaftlichen Eintrittsbarrieren praktisch unangetastet. Mit der hier vorgeschlagenen Regulierungsalternative würden aufgrund der sehr niedrigen Kosten für den Markteintritt (und -austritt) neue, sinnvolle Marktzugangsmöglichkeiten eröffnet. Insbesondere wären die Eintrittsbarrieren für die Entscheidung eines neuen Anbieters über einen Markteintritt völlig irrelevant – vorausgesetzt, der etablierte Betreiber schließt den Zugang Dritter zu seinen Sitzplatzkapazitäten nicht aus. Dies wäre dementsprechend in unserem Regulierungsansatz der zentrale Ansatzpunkt regulatorischen Eingreifens, um einen diskriminierungsfreien Zugang für Wettbewerber zu gewährleisten. In diesem Punkt besteht auch der Unterschied zu den als Analogie herangezogenen Codesharing-Vereinbarungen, denn diese sind freiwillige geschäftsstrategische Vereinbarungen, die es den beteiligten Unternehmen ermöglichen, ihre Gewinne zu maximieren, auch wenn dies zu einer Verminderung des vorhandenen Wettbewerbs, vor allem bei parallelen Diensten, führen kann.

Darüber hinaus würden die Informationsasymmetrien, die eine (wirksamere) Regulierung im Eisenbahnsystem auf herkömmliche Weise regelmäßig erschweren, auf ein Minimum reduziert – d. h. die Ermittlung der (Grenz-)Kosten für das Angebot eines Sitzplatzes in einem bestimmten Zug, um eine wettbewerbswidrige Preisdiskriminierung des etablierten Betreibers zu verhindern. Allerdings ist die Bestimmung eines solchen nichtdiskriminierenden Zugangspreises alles andere als trivial. Alle anderen relevanten Informationen, insbesondere die gesamte erwartete effektive Sitzplatzkapazität eines Zuges, können aber direkt beim etablierten Zugbetreiber eingeholt werden.

Ein weiterer wichtiger komparativer Vorteil des alternativen Regulierungssystems besteht darin, dass es die infrage kommenden Wettbewerber nicht auf andere Eisenbahnunternehmen beschränkt. Es sind auch Newcomer aus ganz anderen Branchen denkbar wie große Konzertkartenhändler (z. B. Eventim), Online-Reisebüros und andere Großhändler von Reisedienstleistungen, die dann in die Lage versetzt würden, ihren Anteil an den Sitzplatzbeständen des etablierten Bahnbetreibers in direktem Wettbewerb zu seinen eigenen Preisen zu vermarkten.

Selbstverständlich sind auch potenzielle Nachteile und Mängel einer Umsetzung des Regulierungsvorschlags zu beachten. Zwar sollten im Gegensatz zum Codeshare keine negativen Auswirkungen auf den Wettbewerb auftreten, doch wird auch der regulierte Zugang zur Sitzplatzkapazität keine neuen oder zusätzlichen Verkehrsdienste auf dem Markt schaffen, da es keine eingebauten Anreize zur Kapazitätserweiterung gibt. Angesichts der völlig unbefriedigenden Kapazitätsauslastung wäre das Modell dennoch ein großer Schritt vorwärts, um die wirtschaftliche Tragfähigkeit des Schienenpersonenfernverkehrs zu erhalten und seine Wettbewerbsfähigkeit zu verbessern.

Darüber hinaus beseitigt der vorgeschlagene innovative Regulierungsansatz nicht die Notwendigkeit, den Zugang zur Infrastruktur zu verbessern, um all jenen Neuankömmlingen, die ihren eigenen Zugverkehr betreiben wollen, eine diskriminierungsfreie Nutzung des bestehenden Schienen- und Bahnhofsnetzes zu ermöglichen. Dementsprechend ist er auch nicht als Ersatz für Open-Access-Regelungen gedacht, sondern als komplementärer Ansatz, der mehrere kleinräumige Zugänge im gesamten Fernverkehrsnetz der Deutschen Bahn ermöglichen würde.

Eine wichtige Lehre aus den Codeshare-Vereinbarungen in der Luftfahrtindustrie ist schließlich die Notwendigkeit, kartellrechtliche Bestimmungen strikt durchzusetzen, um Preisabsprachen zwischen dem etablierten Betreiber und den Unternehmen, die Zugang zu dessen Sitzplatzangebot erhalten haben, zu verhindern.

## Fazit und Schlussfolgerungen

Das hier vorgeschlagene ergänzende Regulierungssystem kann einen einfachen und potenziell sehr effektiven Weg für den Markteintritt in das deutsche Personenfernverkehrssegment bieten. Es vermeidet Abschreckungseffekte, die sich typischerweise aus der Komplexität des bestehenden Regulierungsrahmens für den SPFV ergeben. Die etablierte Betreiberin, die Deutsche Bahn, wäre zwar weiterhin die dominierende Akteurin in diesem Marktsegment, hätte aber dennoch stärkere Anreize als je zuvor für Effizienz und bessere Servicequalität. Mehr Wettbewerb im Vertrieb und eine höhere Kapazitätsauslastung könnten so ohne größere Hindernisse und zu vergleichsweise geringen Regulierungskosten erreicht werden. Die höhere Kapazitätsauslastung könnte auch das wirtschaftliche Ergebnis der etablierten Betreiberin verbessern, sodass Widerstand gegen ein solches Regulierungsregime auf lange Sicht unwahrscheinlich erscheint. Müsste die Deutsche Bahn als (privates) Wirtschaftsunternehmen am Markt bestehen, wäre sie umgekehrt sogar sicher an einer solchen Lösung interessiert. Der aktuelle Nachfragerückgang im SPFV infolge der COVID-19-Pandemie böte eine günstige Gelegenheit, den vorgeschlagenen innovativen Regulierungsansatz auf ausgewählten Hauptstrecken zu erproben.
